# Association between weekend catch-up sleep and specific depressive symptoms: a real world research

**DOI:** 10.3389/fpsyt.2025.1698743

**Published:** 2025-12-03

**Authors:** Siheng Ma, Qilong Wang, Yuyu Zhang, Min Cai, Rui Su, Tingrui Wang, Huaning Wang, Xianyang Wang, Guangtao Hu

**Affiliations:** 1 Department of Psychiatry, Xijing Hospital, The Fourth Military Medical University, Xi’an, Shaanxi, China; 2 Department of Psychiatry, Gansu University of Chinese Medicine, Gansu Provincial People’s Hospital, Lanzhou, Gansu, China; 3 Department of Military Medical Psychology, The Fourth Military Medical University, Xi’an, Shaanxi, China; 4 Department of Psychological Medicine, 958th Hospital, The Third Military Medical University, Chongqing, China

**Keywords:** depressive symptoms, weekend catch-up sleep, sleep disturbance, external validation, NHANES

## Abstract

**Background:**

Workday sleep deprivation has become normalized in contemporary society. While previous research suggested that weekend catch-up sleep (WCS) could reduce the risk of depression, there is currently limited evidence supporting the role of WCS in reducing the development of specific depressive symptoms. Therefore, the objective of this study was to investigate the relationship between WCS and specific depressive symptoms among American.

**Methods:**

A total of 7,695 participants were recruited from the 2017–2020 NHANES. Concurrently, an external validation set comprising 180 independent clinical participants from Xijing Hospital was collected. The presence of depressive symptoms was determined through analysis of the PHQ-9 questionnaire, with each item representing one distinct type of depressive symptom. Multivariate logistic regression and generalized additive models were used to determine the correlation between WCS sleep and specific depressive symptoms. Subgroup analysis was used to reveal differences between WCS and specific depressive symptoms in specific populations.

**Results:**

The findings of the multiple logistic regression analysis indicated that WCS associated with a wide range of specific depressive symptoms, particularly suicidal ideation, with OR = 0.53 (95% CI: 0.33–0.85) in the NHANES group and OR = 0.12 (95% CI: 0.04–0.41) in the clinical sample. Following adjustment for all covariates, non-linear associations of WCS with sleep disturbance and psychomotor disturbance in both samples (NHANES/Xijing Hospital). Furthermore, the results of subgroup analyses indicated that specific subgroups of the vast majority of depressive symptoms were correlated with WCS.

**Conclusions:**

The results of this study confirm that WCS inversely related to specific depressive symptoms, particularly among individuals under 35 with suicidal ideation.

## Introduction

Major depressive disorder (MDD) is a highly prevalent mental health condition, with an estimated 332 million cases reported worldwide. According to the most recent Global Burden of Disease (GBD) report, depression has been identified as the leading cause of global disease burden, surpassing other major health concerns, and is also the second leading cause of years of healthy life lost (1). Depression episodes have been shown to be associated with an increased likelihood of sleep disturbances, changes in appetite, and cognitive dysfunction (2–4). Furthermore, major depressive episodes have been demonstrated to significantly elevate the risk of suicidal ideas and attempts (5). The critical importance of depression severity variations arising from distinct symptom dimensions is under examination. This significance is empirically demonstrated by a cohort study that identified four discrete MDD severity levels based specifically on altered appetite manifestations (6). This finding indicates that MDD has a variety of symptomatic manifestations, and the subtypes and severity levels derived from different symptom dimensions vary as well (7). However, the relevance in symptoms of MDD, the influence factors of the severity, the prognosis of MDD still need more exploration.

Sleep disorder is a grave health concern that has been identified as a risk factor for depression across various age groups (8). The National Sleep Foundation recommends that adults obtain 7–9 hours of sleep on a regular basis (9). However, the contemporary society often presents challenges that hinder adults from achieving sufficient sleep during weekdays, due to factors such as professional obligations, lifestyle choices, and environmental influences (10). Consequently, individuals often seek to compensate for their inadequate weekday sleep by extending their sleep duration on weekends or non-weekdays, a phenomenon referred to as “weekend catch-up sleep” (WCS) (11).

A large number of studies have previously examined the relationship between WCS and depression. For example, these studies primarily focused on DSM-based categorical definitions of depression, and the results suggested the potential for remission of depressive symptoms following WCS (11–13). Although these studies provided valuable insights, they mainly generalized depression as a homogeneous entity and ignored the heterogeneity of patients’ symptoms. According to the DSM-5, there are 227 possible combinations of symptoms (14, 15). Given the heterogeneity of depressive symptoms, it is imperative to shift the research perspective from solely studying diagnostic categories to understanding the relationship between WCS and specific depressive symptoms. This shift in perspective acknowledges the complexity of depression at the clinical level and aims to reveal how depressive symptoms are influenced by WCS, which could supply a potential for the development of more individualized and targeted therapeutic interventions.

A body of research has identified the crucial relationship between WCS and certain depressive symptoms, including suicidal ideation, self-injurious behavior, inattention, and depressed mood (16–18). For instance, Hyunseo Lee’s findings indicated that WCS was associated with suicidal ideation in Korean adolescents, with WCS of less than 1 hour exhibiting the strongest association (16). Seog Ju Kim’s research suggested a link between WCS and inattention, and that an increase in WCS time was significantly associated with more omissions and errors of commission on attentional tasks (17). Furthermore, Wang et al. found that WCS was associated with anxiety and depressed mood, and that longer WCS time was associated with depressive and anxiety symptoms (18). However, the extant studies have not systematically examined multiple depressive symptom clusters, and the present study remedies the shortcomings of the above studies by developing a more detailed discussion.

Although previous studies have explored the relationship between WCS and depression, most have treated depression as a homogeneous entity. Given the symptomatic heterogeneity of MDD, there is a need to examine how WCS relates to specific depressive symptoms. This study aims to address this gap by employing a symptom-centered approach to elucidate the association between WCS and individual depressive symptoms, thereby contributing to more personalized intervention strategies.

## Methods

### Data source and study population

The National Health and Nutrition Examination Survey (NHANES) is a cross-sectional research program designed to assess the health and nutritional status of the population of the U.S. (19). NHANES combines interviews, physical examinations, and laboratory tests to provide a comprehensive view of public health. The survey is conducted on a nationwide scale, employing a complex multistage probability sampling design. The CDC conducts a continuous survey on an annual basis, with data collected and released for public use every two years. For the present study, a specific data cycle (from 2017 through 2020) was extracted, based on the presence or absence of relevant variables for the study’s objectives. The NHANES protocol was reviewed and approved by the Research Ethics Review Board of the National Center for Health Statistics, and all subjects provided written informed consent (https://www.cdc.gov/nchs/nhanes/irba98.htm).

The external validation data were collected in May 2025 at Xijing Hospital, with data from 180 participants. All participants were informed about the study and agreed to share their relevant data anonymously and were recruited in-person at Xijing Hospital. Due to the complexity of the recruitment process, data completion is divided into online and offline completion. The Xijing Hospital sample was a convenience sample without complex sampling design. Therefore, no sampling weights or design adjustments were applied. Recruitment for the Xijing Hospital sample was conducted from May 1 to May 31, 2025, in the outpatient department and inpatient ward of the Psychiatry Department of our hospital. Through the outpatient registration system, potential participants aged ≥ 20 years old who sought medical attention for emotional issues (e.g., depression, anxiety) were screened, and a total of 423 individuals were approached (312 outpatients and 111 inpatients). After researchers explained the study details face-to-face, 239 individuals refused to participate (refusal rate: 56.5%), with the main reasons including time conflicts (128 individuals, 53.6%), privacy concerns (76 individuals, 31.8%), and lack of willingness to participate (35 individuals, 15.6%). A total of 184 individuals initially agreed to participate, all of whom met the inclusion criteria (age ≥ 20 years old, completion of mental health screening, sleep questionnaire, and covariate surveys). Number of 4 participants were excluded, and all 180 individuals were finally included in the analysis. The present study was reviewed and approved by the Ethics Committee of Xijing Hospital. The ethics number is: KY20242298-C-1.

**Figure 1:**
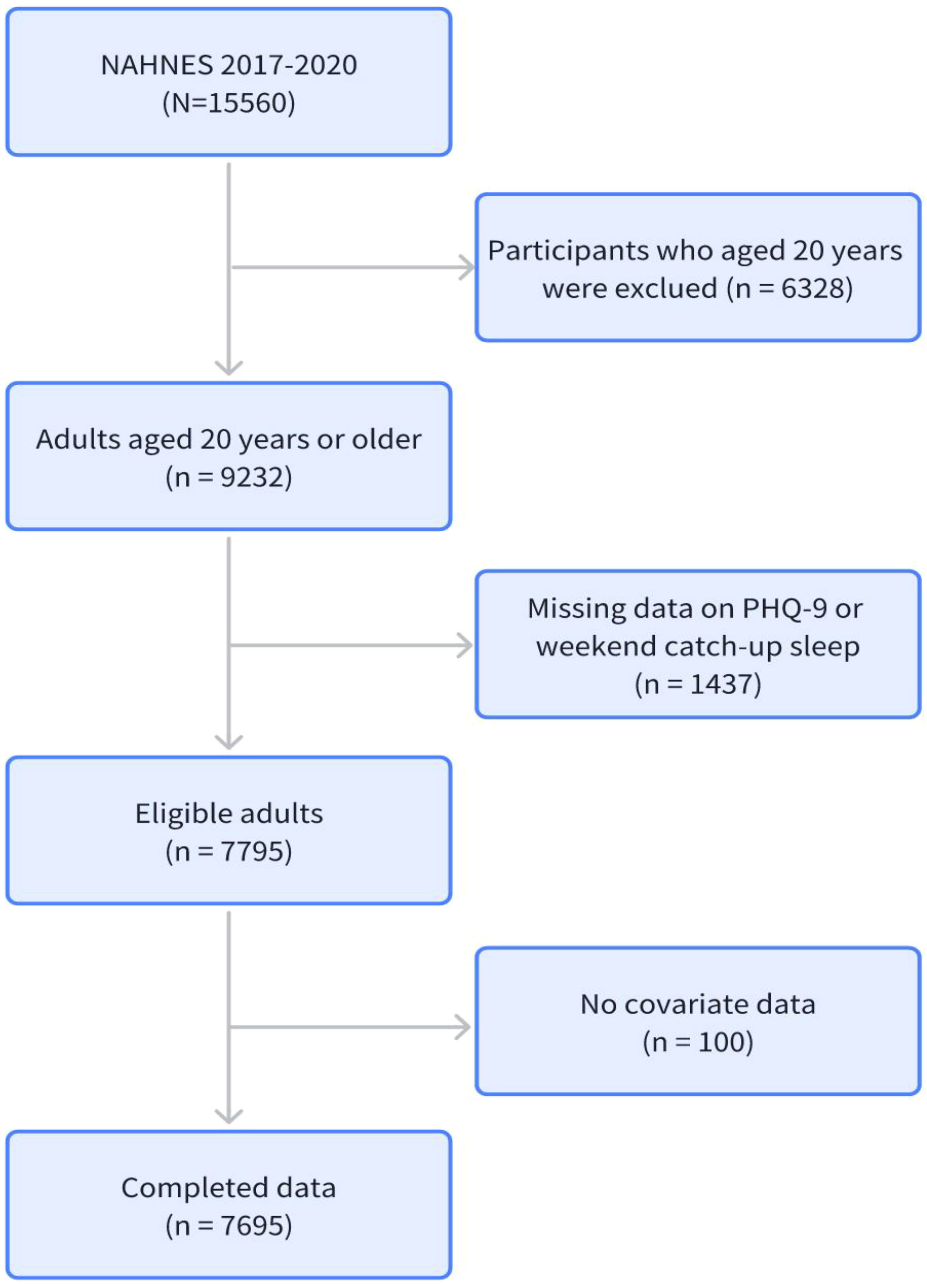
The follow chat of this study.

The inclusion criteria were as follows: first, participants had to be 20 years of age or older; second, participants had to undergo a complete mental health screening; third, participants had to complete a complete sleep questionnaire; and fourth, participants had to complete all covariates. The flow chart illustrating the participant enrollment is presented in **Figure 1**.

The collection of data for the external validation set was conducted in strict accordance with the aforementioned standards. The inclusion criteria were as follows: first, participants had to be 20 years of age or older; second, participants had to undergo a complete mental health screening; third, participants had to complete a complete sleep questionnaire; and fourth, participants had to complete all covariates. **Supplementary Figure 1** presents a flowchart outlining the registration process for external validation participants.

### Evaluation of WCS

The data for WCS evaluation were obtained from the Sleep Disorders Questionnaire. In NHANES 2017-2020, the sleep duration of participants on weekdays and weekends was calculated based on their responses to the following questions: i) “What time do you usually go to sleep on a weekdays or weekends?” and ii) “What time do you usually wake up on weekdays or weekends?” The WCS duration was then calculated by subtracting the weekday sleep time from the weekend sleep time. For the purpose of this study, WCS was defined as an increase in weekend sleep hours relative to weekday sleep hours. Based on this definition, the study population was divided into two groups: a WCS group and a non-WCS group (20). The collection of WCS in external validation data follows the same procedure and is divided into two groups.

### Assessment of depressive symptoms

The PHQ-9 was utilized to evaluate depressive symptoms and levels of depression (21). The PHQ-9 is a self-administered questionnaire that was developed based on the nine diagnostic signs and symptoms of depression outlined in the DSM-5, namely: “Not interested in doing things? (Anhedonia), “Feeling down, depressed, or hopeless? (Depressed mood), “Difficulty falling asleep or sleeping too much?” (Sleep disturbance), “Feeling tired or low energy?” (Fatigue), “Loss of appetite or overeating” (Appetite change), “Feeling bad about yourself” (Feeling bad about self), “Difficulty concentrating on things” (Difficulty concentrating), “Moving or talking slowly or too quickly” (Psychomotor disturbance), and “feeling better off dead” (Suicidal ideation) (22). Each item or symptom dimension of the PHQ-9 constitutes a distinct phenotype, with scores ranging from “0” (not at all) to “3” (almost daily). If the item was scored 2 or above, the specific symptom was considered to be present (23). The PHQ-9 total scores ranged from 0 to 27. Conventional consensus, as established by prior research, posits that the optimal threshold for major depression is a score of 10 of PHQ-9, exhibiting a sensitivity and specificity of 88% (22). Consequently, in this study, the presence of current depression was determined by a PHQ-9 score of ≥ 10 (24). All participants recruited at Xijing Hospital were clinically evaluated by psychiatrists to confirm symptom presence.

### Assessment of covariates

The participants’ demographic and behavioral characteristics were the focus of the extraction process. These characteristics included gender, age, race, and other pertinent data points such as educational level, marital status, smoking status, alcohol drinking status, and leisure time physical activity level. Additionally, body mass index (BMI), a representative indicator of physical examination, was included in the analysis. The age of the participants was categorized into three groups: ≤ 35 years, 36–64 years, and ≥ 65 years. The race of the participants included Mexican American, Other Hispanic, Non-Hispanic White, Non-Hispanic Black, and Other, as categorized in the NHANES questionnaire. Due to the presence of substantial disparities in ethnic distribution across mainland China, researchers opted to exclude this covariate in order to mitigate the potential impact of ethnicity or race on the results of the external validation dataset. The educational attainment of the participants was categorized into three categories: less than high school, high school or equivalent, and college or above. Marital status was categorized as follows: married/living with partner, widowed/divorced/separated, and never married. The smoking status of each participant was ascertained and classified as either never smoking or smoking through a home interview. The consumption of more than five alcoholic beverages per day on average during the 12 months prior to the interview was considered current excessive alcohol usage. Leisure time physical activity level was categorized into three classes based on the frequency from moderate to vigorous exercise: 0, 1-2, and ≥3 times/week (25). Additionally, obesity was defined as a BMI of ≥ 30 kg/m^2^.

### Statistical analysis

Given the complex stratified multistage probability sampling design utilized by NHANES, the consideration of sample weights, strata, and primary sampling units (PSUs) was deemed essential to address the issue of over-sampling of minority groups, in accordance with established guidelines. Continuous variables were presented as mean (standard error, SE) and/or median (interquartile range, IQR). Categorical variables were presented as frequencies and percentages. A chi-square test (Chi-square tests) was performed on the survey data to compare the differences between the non-WCS group and the WCS group.

Multivariate logistic regression was employed to explore the association between WCS and specific depressive symptoms. Three models were constructed: in the crude model 1, no adjustment was made for any covariates; in Model 2 adjustments were made for age and race; and in Model 3, adjustments were made for age + race + educational level + smoking status. Meanwhile, we implemented the Bonferroni correction for *P*-value adjustment (establishing a revised significance threshold of α = 0.05/9≈0.0056). Generalized additive modeling (GAM) was used to examine the nonlinear relationship between WCS and specific depressive symptoms. Subgroup analysis was used to reveal differences between WCS and specific depressive symptoms in specific populations.

The data from the external validation set were statistically described using the same methods. Continuous variables were expressed as mean (standard error, SE) and/or median (interquartile range, IQR). Categorical variables were expressed as frequencies and percentages. A Chi-square tests were conducted to assess the disparities between the non-WCS group and the WCS group. Multivariate logistic regression analysis, generalized additive models, and subgroup analysis were all applied to the external validation data to verify the consistency of the results. To quantify our capacity to detect true effects given the observed parameters, *post-hoc* power analyses were conducted. With the achieved sample size (n = 180), significance threshold (α = 0.05), statistical power reached 90% for primary outcomes. This exceeds the 80% minimum threshold recommended by Cohen, indicating robust inferential reliability. For the core outcome indicators (suicidal ideation), we used the E-value (a sensitivity index for exposure-outcome associations) to assess the degree of interference from unmeasured confounding factors on the results (26).

All analyses were performed using the EmpowerStats software and R Statistical Software Version 4.4.2 (R Foundation for Statistics, http://www.r-project.org). The statistical significance of all two-tailed tests was set as a critical value of 0.05.

## Results

### Population characteristics

**Table 1** lists the basic characteristics of participants in the NHANES and Xijing hospital. A significant discrepancy was observed in the prevalence of depressive symptoms between the two groups. The proportion of patients with depressive symptoms in the Xijing Hospital group who were not in WCS status (46.34%) was considerably higher than that in the NHANES group (9.83%). A similar trend was observed in WCS status, where the detection rate of depressive symptoms in the Xijing Hospital group (25.9%) was also significantly higher than that in the NHANES group (7.86%). The two groups showed significant statistical differences in age, smoking status, leisure time physical activity level, and current depressive symptoms. There were significant statistical differences in alcohol drinking status among participants in the external validation dataset, but not among NHANES participants. It is worth noting that the external validation dataset did not include participants aged 65 and above, possibly due to the data collection method. In addition, a notable variation in the allocation of diverse variables was observed across participants in the two groups. The obesity rate in the Non-WCS group of the external validation dataset was 0%, while only 4 people (2.88%) in the WCS group were obese. This distribution differs significantly from that of NHANES. A similar tendency is observed in the context of marital status.

**Table 1:** Baseline characteristics of participants.

Variables	NHANES		Xijing hospital	
	Non WCS (n = 4170)	WCS (n = 3525)	Non WCS (n = 41)	WCS (n = 139)
Gender				
Men	2064 (49.50)	1700 (48.23)	19 (46.34)	52 (37.41)
Women	2106 (50.50)	1825 (51.77)	22 (53.66)	87 (62.59)
Age				
≤35	967 (23.19)	944 (26.78)	20 (48.78)	101 (72.66)
36-64	1983 (47.55)	1922 (54.52)	21 (51.22)	38 (27.34)
≥65	1220 (29.26)	659 (18.70)		
Race			/	/
Mexican American	442 (10.60)	456 (12.94)		
Other Hispanic	427 (10.24)	360 (10.21)		
Non-Hispanic White	1615 (38.73)	1127 (31.97)		
Non-Hispanic Black	1062 (25.47)	963 (27.32)		
Other Race	624 (14.96)	619 (17.56)		
Educational level				
Less than high school	723 (17.34)	613 (17.39)	2 (4.88)	7 (5.04)
High school or equivalent	1033 (24.77)	827 (23.46)	3 (7.32)	8 (5.76)
College or above	2414 (57.89)	2085 (59.15)	36 (87.80)	124 (89.21)
Marital status				
Married/living with partner	2433 (58.35)	2070 (58.72)	23 (56.10)	62 (44.60)
Widowed/divorced/separated	949 (22.76)	748 (21.22)	0 (0.00)	4 (2.88)
Never married	788 (18.90)	707 (20.06)	18 (43.90)	73 (52.52)
Obesity (BMI≥30kg/m^2^)				
No	2360 (56.59)	2010 (57.02)	41 (100.0)	135 (97.12)
Yes	1810 (43.41)	1515 (42.98)	0 (0.00)	4 (2.88)
Alcohol drinking status				
No	3822 (91.65)	3243 (92.00)	23 (56.10)	107 (76.98)
Yes	348 (8.35)	282 (8.00)	18 (43.90)	32 (23.02)
Smoking status				
No	2220 (53.24)	2229 (63.23)	28 (68.29)	115 (82.73)
Yes	1950 (46.76)	1296 (36.77)	13 (31.71)	24 (17.27)
Leisure time physical activity level				
0 times/week	284 (6.81)	273 (7.74)	7 (17.07)	43 (30.94)
1–2 times/week	2926 (70.17)	2377 (67.43)	19 (46.34)	74 (53.24)
≥3 times/week	960 (23.02)	875 (24.82)	15 (36.59)	22 (15.83)
Depressive symptoms				
No	3760 (90.17)	3248 (92.14)	22 (53.66)	103 (74.10)
Yes	410 (9.83)	277 (7.86)	19 (46.34)	36 (25.90)

BMI, Body mass index.

### Association between WCS and specific depressive symptoms

**Table 2** (NHANES) presents the correlation between WCS and specific depressive symptoms. In Model 1 (unadjusted for any variables), with the exception of fatigue and appetite changes, which demonstrated no significant differences in WCS, all other symptoms exhibited statistically significant differences in WCS, and the effect was most evident for suicidal ideation (OR = 0.507, 95% CI: 0.307-0.838, *P* = 0.00803). Model 2 was adjusted for age and race as covariates. The results remained consistent with Model 1, and the effect against suicidal ideation remained the most significant (OR = 0.514, 95% CI: 0.310-0.853, *P* = 0.00998). In Model 3, further adjustments were made for education level and smoking status. The effect of WCS on anhedonia and psychomotor disturbance changed from significant to insignificant, while its effect on fatigue and appetite changes remained insignificant (*P* > 0.05).

**Table 2:** Multivariable logistic regression analyses for WCS and specific depression symptoms. (NHANES).

Variables	NHANES					
	Model 1		Model 2		Model 3	
	OR	*P*	OR	*P*	OR	*P*
Anhedonia	0.840 (0.716, 0.985)	0.03205	0.829 (0.706, 0.974)	0.02236	0.870 (0.740, 1.023)	0.09151
Depressed mood	0.806 (0.679, 0.957)	0.01387	0.788 (0.663, 0.937)	0.00692	0.824 (0.693, 0.981)	0.0293
Sleep disturbance	0.834 (0.738, 0.942)	0.00355	0.831 (0.735, 0.940)	0.00325	0.869 (0.767, 0.983)	0.02624
Fatigue	0.900 (0.799, 1.014)	0.08243	0.897 (0.796, 1.012)	0.0772	0.930 (0.824, 1.050)	0.24141
Appetite change	0.973 (0.838, 1.130)	0.71841	0.968 (0.832, 1.125)	0.66866	1.004 (0.862, 1.168)	0.96357
Feeling bad about self	0.725 (0.588, 0.893)	0.00251	0.713 (0.578, 0.881)	0.00168	0.745 (0.603, 0.921)	0.00656
Difficulty concentrating	0.814 (0.675, 0.982)	0.03158	0.792 (0.655, 0.956)	0.01531	0.826 (0.683, 0.999)	0.0484
Psychomotor disturbance	0.755 (0.597, 0.954)	0.0185	0.751 (0.593, 0.951)	0.01762	0.793 (0.626, 1.006)	0.05586
Suicidal ideation	0.507 (0.307, 0.838)	0.00803	0.514 (0.310, 0.853)	0.00998	0.536 (0.323, 0.892)	0.01629

Model 1: unadjusted; Model 2: Model 1 + age and race; Model 3: Model 2 + educational level and smoking status.

OR, odds Ratio.

**Table 3** (Xijing Hospital) presents the association between WCS and specific depressive symptoms. The results obtained from Models 1 through 3 exhibited a high degree of consistency. The six primary symptoms of anhedonia, depressive mood, feeling bad about self, difficulty concentrating, psychomotor disturbance, and suicidal ideation were all closely related to WCS and demonstrated significant statistical differences, and suicidal ideation was the most significant factor (OR = 0.12, 95% CI: 0.035-0.411, *P* = 0.00075). However, it is noteworthy that feeling bad about self exhibited statistical disparities exclusively in Model 3 (OR = 0.259, 95% CI: 0.093-0.720, *P* = 0.00967).

**Table 3:** Multivariable logistic regression analyses for WCS and specific depression symptoms. (Xijing Hospital).

Variables	Xijing hospital					
	Model 1		Model 2		Model 3	
	OR	*P*	OR	*P*	OR	*P*
Anhedonia	0.380 (0.176, 0.820)	0.01368	0.312 (0.138, 0.704)	0.00503	0.235 (0.096, 0.574)	0.00147
Depressed mood	0.375 (0.154, 0.915)	0.03109	0.330 (0.131, 0.836)	0.01934	0.246 (0.090, 0.673)	0.00634
Sleep disturbance	0.609 (0.294, 1.263)	0.18264	0.594 (0.281, 1.255)	0.17204	0.603 (0.276, 1.318)	0.20493
Fatigue	0.779 (0.371, 1.638)	0.51029	0.753 (0.352, 1.614)	0.46652	0.597 (0.262, 1.360)	0.21958
Appetite change	1.471 (0.563, 3.844)	0.43039	1.206 (0.449, 3.239)	0.70986	1.243 (0.449, 3.440)	0.67568
Feeling bad about self	0.398 (0.158, 1.002)	0.05056	0.343 (0.131, 0.897)	0.02917	0.259 (0.093, 0.720)	0.00967
Difficulty concentrating	0.380 (0.176, 0.820)	0.01368	0.343 (0.154, 0.764)	0.00883	0.282 (0.121, 0.662)	0.00361
Psychomotor disturbance	0.330 (0.138, 0.791)	0.01292	0.282 (0.113, 0.705)	0.00674	0.211 (0.079, 0.563)	0.00192
Suicidal ideation	0.186 (0.060, 0.573)	0.00339	0.158 (0.049, 0.509)	0.00202	0.120 (0.035, 0.411)	0.00075

Model 1: unadjusted; Model 2: Model 1 + age and race; Model 3: Model 2 + educational level and smoking status.

OR, odds Ratio.

#### Non−linear relationship between WCS and specific depressive symptoms

**Figure 2:**
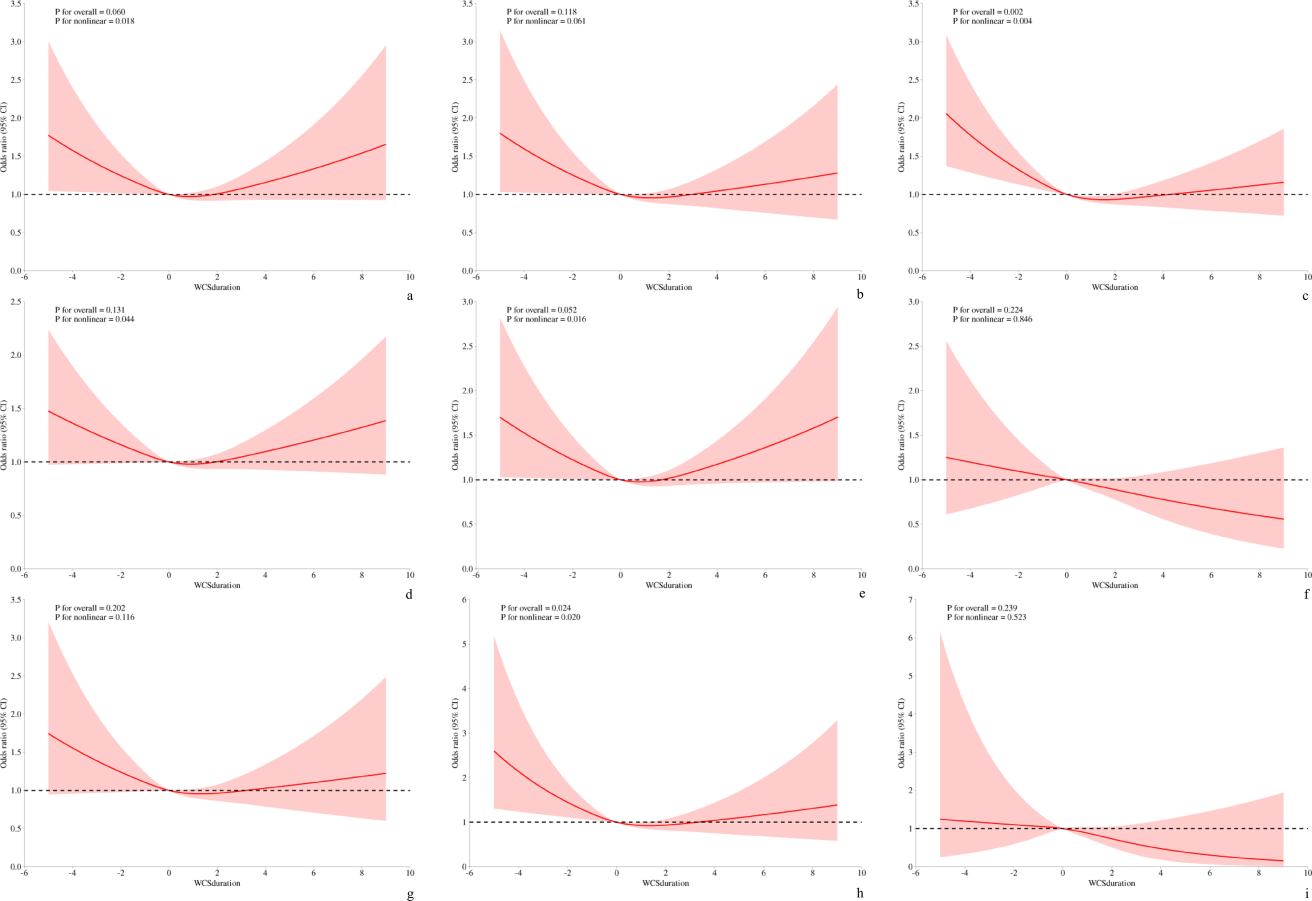
The non-linear relationship between WCS and specific depressive symptom. (NHANES). **(a)** Anhedonia; **(b)** Depressed mood; **(c)** Sleep disturbance; **(d)** Fatigue; **(e)** Appetite change; **(f)** Feeling bad about self; **(g)** Difficulty concentrating; **(h)** Psychomotor disturbance; **(i)** Suicidal ideation.

**Figure 3:**
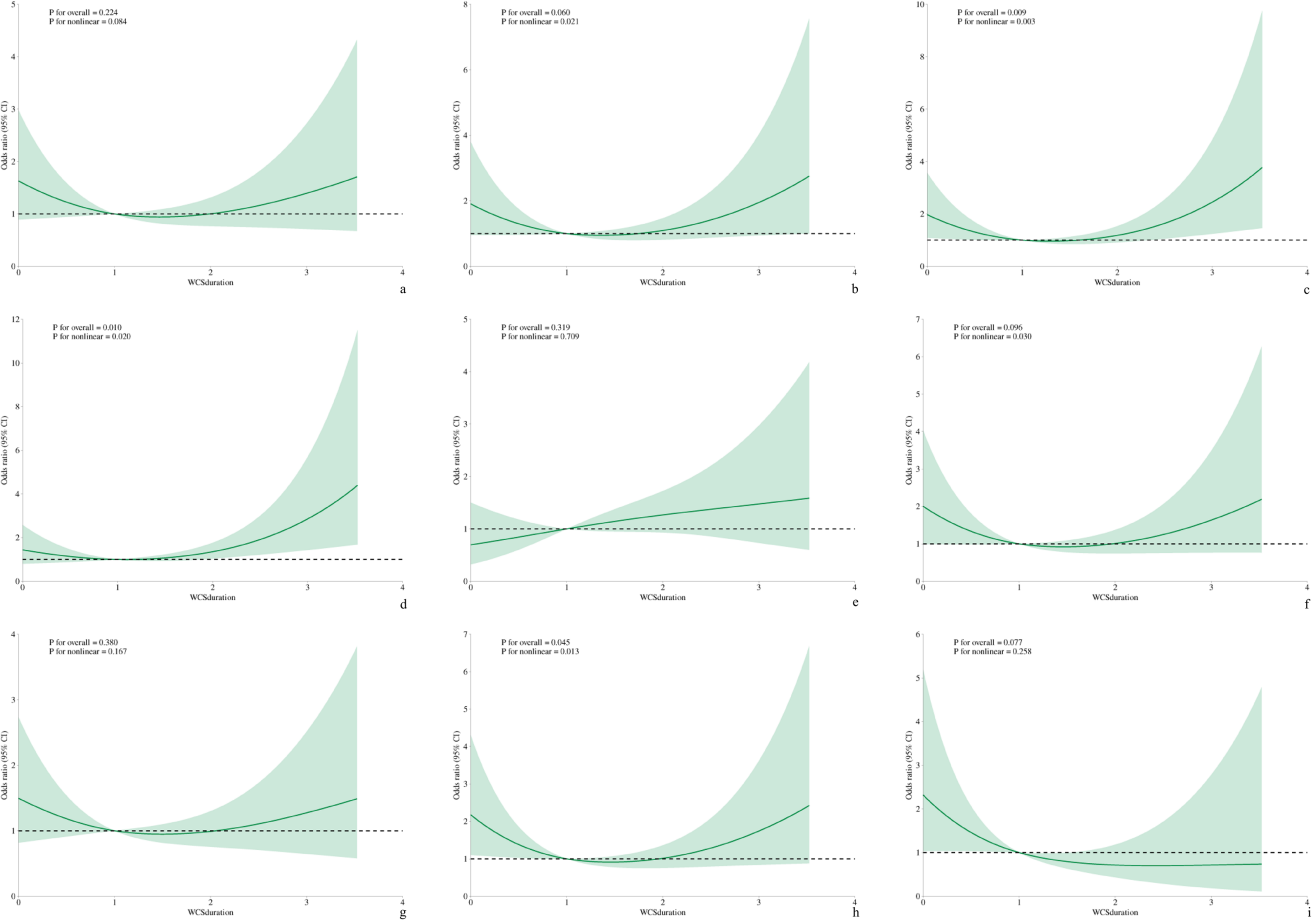
The non-linear relationship between WCS and specific depressive symptom. (Xijing Hospital). **(a)** Anhedonia; **(b)** Depressed mood; **(c)** Sleep disturbance; **(d)** Fatigue; **(e)** Appetite change; **(f)** Feeling bad about self; **(g)** Difficulty concentrating; **(h)** Psychomotor disturbance; **(i)** Suicidal ideation.

The generalized additive model was employed to graphically examine the potential associations between WCS and specific depressive symptoms. **Figures 2**, **3** show the GAM for NHANES and Xijing Hospital, respectively. Following adjustment for all covariates, sleep disturbance and psychomotor disturbance were found to be a non-linear association with WCS in both samples (NHANES/Xijing Hospital, *P*<0.05). Furthermore, the relationship between fatigue and WCS in the Xijing Hospital group exhibited a non-linear association (*P*<0.05), which was not observed in the NHANES. No statistically significant variations were observed in the remaining symptoms.

#### Subgroup analyses

The **Supplementary Materials** contain all subgroup analysis figures. Among all subgroups, only the smoking subgroup in NHANES demonstrated a significant interaction between WCS and anhedonia (*P* < 0.05), while no interaction was observed in the remaining results. The subgroup analysis results from Xijing Hospital demonstrated that in non-smokers aged 35 years or younger, WCS exhibited a significant association with anhedonia, depressive mood, self-directed negative emotions, difficulty concentrating, psychomotor disturbance, and suicidal ideation.

## Discussion

The objective of this study was to examine the relationship between WCS and specific depressive symptoms. The findings from the multivariate logistic regression analysis conducted by NHANES and Xijing Hospital indicate that a one-unit increase in WCS is associated with a significant reduction in the probability of suicidal ideation, with estimates of 47% and 88%, respectively. Furthermore, in the Xijing Hospital group, the effect of WCS exceeded 75% in all cases (*P*<0.05). These findings revealed important implications for the future direction of clinical precision medicine. Furthermore, the results of subgroup analyses indicated that specific subgroups of the vast majority of depressive symptoms were correlated with WCS. These findings underscore the necessity of considering demographic differences when examining the association between depression and WCS.

Patients exhibiting elevated levels of suicidal ideation (SI) demonstrate a heightened propensity for suicide attempts or suicidal behavior. Consequently, SI is regarded as a pivotal clinical indicator that necessitates meticulous observation (27). A meta-analysis of relevant studies suggested a positive correlation between sleep disorders, particularly nightmares and insomnia, and suicidal ideation and behavior in depressed patients (28). A study of gender differences in suicidal ideation revealed that female patients with major depressive disorder experienced higher rates of difficulty in falling asleep and maintaining sleep, compared to male patients with SI (29). A separate study of sleep and suicide in adolescents noted significant sleep abnormalities in a sample of adolescents hospitalized for suicidal crises (30). In summary, there is a need to address sleep disorders when dealing with suicidality in patients with MDD. Sleep deprivation has been demonstrated to result in an augmented inflammatory response, which, in turn, has been shown to affect the psychological state. Research has indicated that chronic sleep deprivation can lead to an increase in pro-inflammatory cytokines, such as tumor necrosis factor-alpha (TNF-alpha) and interleukin-6 (IL-6), which may play a pivotal role in the development of suicidal ideation and behavior (31). In response to the literature recommendations, the present study included correlation and subgroup analyses. The analysis revealed an inverse association between WCS and suicidal ideation probability, with observed probabilities reduced to 12% (88% reduction relative to baseline) and 53% (47% reduction) in exposed cohorts. However, gender-specific subgroup analyses yielded a contradictory conclusion, indicating that men exhibited statistically significant differences after experiencing WCS. One potential explanation for the gender difference may be the influence of sex hormones, such as estrogen and progesterone (32).

Research on the subjects with sleep disorders and anhedonia has demonstrated that sleep deprivation did not result in an improvement of anhedonia in general. Furthermore, improvements in anhedonia were not significant even after sleep restoration (33). This finding is consistent with the results of this research, which demonstrated that sleep restoration, whether with or without sleep, did not improve anhedonia. It has been demonstrated by other studies that the presence and severity of anhedonia, as a core symptom of depression, was significantly associated with more complex depressive symptom presentation and severity (34, 35). The association of anhedonia with sleep disorders may be attributable to the interaction of dysregulated dopaminergic systems and neuroinflammatory pathways. Basic research suggests that reduced activity of the limbic dopamine pathway in the midbrain may result in impaired reward processing, which, in turn, may affect the regulation of the sleep-wake cycle, especially the stability of non-rapid eye movement (NREM) sleep (36). Furthermore, elevated levels of chronic inflammatory factors (e.g., IL-6, TNF-α) have been shown to exacerbate circadian rhythm disturbances by activating the hypothalamic-pituitary-adrenal (HPA) axis. This, in turn, can result in a protracted sleep latency or a reduction in slow-wave sleep (37). In summary, in order to enhance anhedonia beyond the scope of merely addressing sleep disturbances, a more comprehensive and systematic antidepressant treatment is imperative.

A study revealed a correlation between the severity of fatigue and the severity of depression in depressed patients. The study also identified excessive sleep as a significant risk factor for fatigue (38). Furthermore, it has been proposed that the symptom of fatigue persisted in patients with major depressive disorder, even following successful treatment (39, 40). Prolonged fatigue has been shown to induce a chronic stress response, which has been demonstrated to increase cortisol secretion. This, in turn, has been observed to affect the functioning of the HPA axis and further exacerbate depressive symptoms (41). Subjects experiencing fatigue have been shown to exhibit an imbalance in neurotransmitters, particularly a decrease in serotonin (5-HT) and dopamine levels. These neurotransmitters play a pivotal role in regulating mood and cognitive functioning (42). The present study concluded that catch-up sleep was a protective factor that adequately alleviated the symptoms of exhaustion in depressed patients, which are contrary to previous studies. The possible reason for this inconsistency is that the present study did not carefully categorize catch-up sleep time. If catch-up sleep time greater than 1 hour was defined as excessive sleep, then the results could be consistent with those of previous studies. The most recent study addressed this limitation by demonstrating that individuals who augmented their sleep by 1–2 hours exhibited a substantial decrease in the likelihood of developing depression (OR = 0.74, 95% CI: 0.55-0.99). This adjustment resulted in a 26% reduction in risk compared to those who did not supplement their sleep (43).

Our findings of elevated inflammatory markers in acute-phase psychiatric inpatients align with Patten et al. (44), who demonstrated that clinical samples with major depression exhibit distinct physiological profiles (attributed to overrepresentation of severe symptoms) compared to community populations, reinforcing the utility of clinical samples for detecting biologically meaningful signals. In contrast to Ford et al.’s NHANES-derived finding of a modest association between depression and C-reactive protein (CRP; OR = 1.64) in the general population (45), our clinical sample showed a 3.2-fold higher odds ratio for elevated CRP in participants with comorbid anxiety; this discrepancy is consistent with Robinson et al. (46), who noted that clinical samples enrich for more severe phenotypes with greater biological perturbations. Our novel identification of gender-specific biomarker patterns (e.g., higher norepinephrine levels in male patients) extends recent UK Biobank findings of significant differences in psychiatric genetic risk scores (PRS) between clinical and population-based samples, suggesting phenotype-dependent biological associations. Regarding generalizability, NHANES uses a complex stratified probability design to represent the U.S. civilian noninstitutionalized population (with oversampling of racial/ethnic minorities and older adults), whereas our acute inpatient sample relied on convenience sampling—introducing selection bias toward severe, treatment-seeking individuals that likely explains our higher prevalence of severe depressive symptoms (31% vs. NHANES’s 9% in community-dwelling older adults). Consistent with Wells et al. (47), our sample underrepresents older adults (mean age 8 years younger) and racial minorities (22% non-White vs. NHANES’s 34%), limiting direct generalizability but enhancing internal validity for studying acute-phase pathophysiology. While our controlled clinical setting enabled standardized biomarker measurements unavailable in NHANES’s mobile examination centers, a recent Science study notes that clinical prediction models from inpatient settings rarely maintain accuracy in community samples due to comorbidity and treatment history differences; thus, our findings are most applicable to similar acute-care populations, with cautious extrapolation to community settings requiring symptom severity adjustment.

The present study demonstrated a robust correlation between an augmentation in WCS and a substantial alleviation of depressive symptoms. Previous research has indicated that depression is profoundly linked to neurotransmitter imbalances, particularly fluctuations in the levels of 5-hydroxytryptamine (5-HT) and dopamine (DA) (48). The provision of enhanced sleep and opportunities for relaxation by WCS has been demonstrated to facilitate the restoration of optimal neurotransmitter function, thereby leading to a reduction in depressive symptoms. For instance, restorative sleep has been shown to promote the synthesis of 5-HT, a neurotransmitter believed to play a pivotal role in enhancing mood (49). In contrast to individuals lacking WCS, patients experiencing WCS demonstrated enhanced emotional regulation, influencing their coping mechanisms with stress and negative emotions.

Another significant mechanism of WCS may be associated with increased social support and self-adjustment. Research conducted thus far indicates that maintaining an optimal work-life balance contributes to the enhancement of an individual’s social support network (50). In comparison with patients exhibiting elevated levels of stress and inadequate WCS, those demonstrating suitable WCS generally have greater access to emotional support and mutual assistance. This can effectively mitigate depressive symptoms. Moreover, enhanced self-regulation is identified as a pivotal approach for addressing depression (51). The ability to effectively manage emotions and stress has been demonstrated to result in a notable reduction in depressive symptoms.

This study has several significant strengths. First, the study benefited from a large and diverse sample and rigorous statistical adjustments, which increased the generalizability and robustness of the findings. Second, the relationship between specific depressive symptoms and WCS was examined at the symptom level. Thirdly, we examined the relationship between WCS and specific depressive symptoms in specific subgroups. Furthermore, the implementation of GAM have unveiled nonlinear associations between WCS and specific depressive symptoms.

This study is subject to several limitations. Firstly, the cross-sectional design of the study precludes the drawing of causal conclusions. Longitudinal studies are necessary to elucidate the causality and directionality of depression-WCS relationship. Secondly, although we adjusted for many confounders, unmeasured variables may still affect our results. These include diseases such as rhinitis, pregnancy, and chronic stress (52–54). This study carries the potential risk of unmeasured confounding factors. Physiological factors such as chronic rhinitis and thyroid dysfunction, as well as psychosocial factors such as childhood trauma and chronic stress, may interfere with the association between WCS and depressive symptoms (55–58). Although the robustness of the results has been verified through E-value analysis and propensity score matching, the impact of such factors cannot be completely ruled out. Future studies can adopt a prospective cohort design and include multi-dimensional physiological indicators (e.g., thyroid function, inflammatory factor panels), objective psychological assessment tools (e.g., the Childhood Trauma Questionnaire), and sleep monitoring data (e.g., polysomnography) to further control confounding and improve the ability of causal inference. Thirdly, both the NHANES and Xijing Hospital data relied on self-reported measures for sleep and depressive symptoms, which may introduce recall bias or social desirability bias. To enhance consistency, we used standardized instruments (PHQ-9 and sleep questionnaires) across both samples. However, cultural and contextual differences between the U.S. and Chinese populations may affect self-reporting patterns. Future studies should incorporate objective measures such as actigraphy to complement self-reported data. We acknowledge the limitations in generalizability due to: The NHANES sample vs. the Xijing Hospital sample; Differences in sampling methods (population-based vs. hospital-based); Demographic and clinical severity differences (higher depression rates in the Xijing sample). Last, While clinically enriched the external validation sample’s modest size and potential lack of full representativeness warrant acknowledgment. The inclusion rate of the Xijing Hospital sample was 42.6% (180/423), which is lower than that of population-based sampling studies such as NHANES. The main reasons include the high level of privacy concern among psychiatric patients (31.8% of refusers declined due to privacy concerns) and the tight schedule of outpatient consultations (53.6% refused due to time conflicts). This inclusion rate is generally consistent with that of similar domestic and international studies using convenience samples from psychiatric outpatient settings (inclusion rates usually range from 35% to 50%). In the future, we will endeavor to validate our results using more reliable and accurate sleep data based on objective measures and expand our validation samples.

## Conclusions

The present study identified associations between WCS and specific depressive symptoms, and its divergent effect in different subgroups. WCS has a significant effect on the vast majority of depressive symptoms, especially suicidal ideation. Nonlinear associations were identified between WCS and sleep disturbances and psychomotor disorder. However, the cross-sectional design of the study did not allow for the establishment of causality. Consequently, the necessity for further longitudinal studies, particularly those that seek to delve deeper into the mechanisms underpinning the observed associations, is paramount. These endeavors are essential for replicating these findings and ascertaining the directionality and causality of the associations. The elucidation of the depression-sleep relationship at the symptom level stands to significantly advance the development of precision medicine in the realm of mental health.

## Data Availability

The original contributions presented in the study are included in the article/**Supplementary Material**. Further inquiries can be directed to the corresponding author.
