# Supplementary material for: Association between weekend catch-up sleep and specific depressive symptoms: a real world research
Source: Front Psychiatry. 2025 Dec 3;16:1698743. doi: 10.3389/fpsyt.2025.1698743 (PMC12708574; doi:10.3389/fpsyt.2025.1698743)
Supplement: Supplementary file 1 [file DataSheet1.pdf]

Variables	n (%)	Non WCS	WCS	OR (95%CI)	P	P for interaction
		No. of events/ No. of total				
All patients	7695 (100.00)	394/4170	284/3525	0.84 (0.72 ~ 0.99)	0.032	
Gender						0.890
Men	3764 (48.91)	182/2064	126/1700	0.83 (0.65 ~ 1.05)	0.118	
Women	3931 (51.09)	212/2106	158/1825	0.85 (0.68 ~ 1.05)	0.132	
Age						0.652
≤ 35	1911 (24.83)	94/967	73/944	0.78 (0.57 ~ 1.07)	0.125	
≥ 65	1879 (24.42)	104/1220	54/659	0.96 (0.68 ~ 1.35)	0.806	
36-64	3905 (50.75)	196/1983	157/1922	0.81 (0.65 ~ 1.01)	0.062	
Smoke						0.018
No	4449 (57.82)	179/2220	133/2229	0.72 (0.57 ~ 0.91)	0.006	
Yes	3246 (42.18)	215/1950	151/1296	1.06 (0.85 ~ 1.33)	0.581	

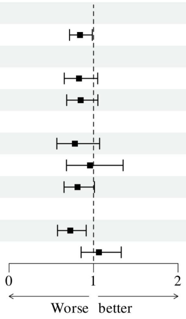

A) Anhedonia

Variables	n (%)	Non WCS	WCS	OR (95%CI)	P	P for interaction
		No. of events/ No. of total				
All patients	7695 (100.00)	345/4170	239/3525	0.81 (0.68 ~ 0.96)	0.014	
Gender						0.884
Men	3764 (48.91)	151/2064	100/1700	0.79 (0.61 ~ 1.03)	0.080	
Women	3931 (51.09)	194/2106	139/1825	0.81 (0.65 ~ 1.02)	0.074	
Age						0.032
≤ 35	1911 (24.83)	84/967	53/944	0.63 (0.44 ~ 0.89)	0.010	
≥ 65	1879 (24.42)	81/1220	52/659	1.20 (0.84 ~ 1.73)	0.313	
36-64	3905 (50.75)	180/1983	134/1922	0.75 (0.59 ~ 0.95)	0.016	
Smoke						0.648
No	4449 (57.82)	149/2220	123/2229	0.81 (0.63 ~ 1.04)	0.097	
Yes	3246 (42.18)	196/1950	116/1296	0.88 (0.69 ~ 1.12)	0.298	

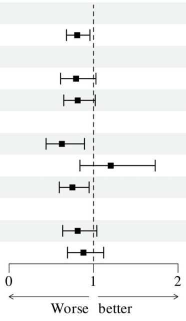

B) Depressed mood

Variables	n (%)	Non WCS	WCS	OR (95%CI)	P	P for interaction
		No. of events/ No. of total				
All patients	7695 (100.00)	730/4170	530/3525	0.83 (0.74 ~ 0.94)	0.004	
Gender						0.775
Men	3764 (48.91)	327/2064	234/1700	0.85 (0.71 ~ 1.02)	0.075	
Women	3931 (51.09)	403/2106	296/1825	0.82 (0.69 ~ 0.96)	0.017	
Age						0.004
≤ 35	1911 (24.83)	186/967	125/944	0.64 (0.50 ~ 0.82)	<.001	
≥ 65	1879 (24.42)	190/1220	116/659	1.16 (0.90 ~ 1.49)	0.256	
36-64	3905 (50.75)	354/1983	289/1922	0.81 (0.69 ~ 0.97)	0.018	
Smoke						0.645
No	4449 (57.82)	321/2220	280/2229	0.85 (0.72 ~ 1.01)	0.064	
Yes	3246 (42.18)	409/1950	250/1296	0.90 (0.76 ~ 1.07)	0.243	

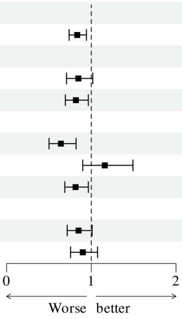

C) Sleep disturbance

Variables	n (%)	Non WCS	WCS	OR (95%CI)	P	P for interaction
		No. of events/ No. of total				
All patients	7695 (100.00)	750/4170	581/3525	0.90 (0.80 ~ 1.01)	0.082	
Gender						0.591
Men	3764 (48.91)	319/2064	247/1700	0.93 (0.78 ~ 1.11)	0.429	
Women	3931 (51.09)	431/2106	334/1825	0.87 (0.74 ~ 1.02)	0.088	
Age						0.038
≤ 35	1911 (24.83)	202/967	145/944	0.69 (0.54 ~ 0.87)	0.002	
≥ 65	1879 (24.42)	206/1220	109/659	0.98 (0.76 ~ 1.26)	0.848	
36-64	3905 (50.75)	342/1983	327/1922	0.98 (0.83 ~ 1.16)	0.847	
Smoke						0.095
No	4449 (57.82)	355/2220	310/2229	0.85 (0.72 ~ 1.00)	0.051	
Yes	3246 (42.18)	395/1950	271/1296	1.04 (0.88 ~ 1.24)	0.651	

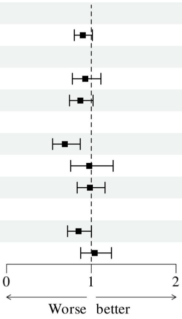

D) Fatigue

Variables	n (%)	Non WCS	WCS	OR (95%CI)	P	P for interaction
		No. of events/ No. of total				
All patients	7695 (100.00)	422/4170	348/3525	0.97 (0.84 ~ 1.13)	0.718	
Gender						0.713
Men	3764 (48.91)	184/2064	143/1700	0.94 (0.75 ~ 1.18)	0.586	
Women	3931 (51.09)	238/2106	205/1825	0.99 (0.81 ~ 1.21)	0.946	
Age						0.102
≤ 35	1911 (24.83)	107/967	94/944	0.89 (0.66 ~ 1.19)	0.430	
≥ 65	1879 (24.42)	109/1220	75/659	1.31 (0.96 ~ 1.79)	0.089	
36-64	3905 (50.75)	206/1983	179/1922	0.89 (0.72 ~ 1.09)	0.260	
Smoke						0.062
No	4449 (57.82)	203/2220	181/2229	0.88 (0.71 ~ 1.08)	0.224	
Yes	3246 (42.18)	219/1950	167/1296	1.17 (0.94 ~ 1.45)	0.154	

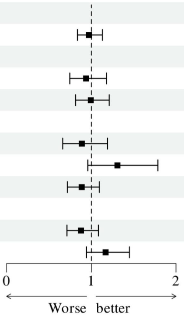

E) Appetite change

Variables	n (%)	Non WCS	WCS	OR (95%CI)	P	P for interaction
		No. of events/ No. of total				
All patients	7695 (100.00)	241/4170	150/3525	0.72 (0.59 ~ 0.89)	0.003	
Gender						0.034
Men	3764 (48.91)	119/2064	56/1700	0.56 (0.40 ~ 0.77)	<.001	
Women	3931 (51.09)	122/2106	94/1825	0.88 (0.67 ~ 1.16)	0.378	
Age						0.821
≤ 35	1911 (24.83)	64/967	42/944	0.66 (0.44 ~ 0.98)	0.040	
≥ 65	1879 (24.42)	68/1220	26/659	0.70 (0.44 ~ 1.10)	0.124	
36-64	3905 (50.75)	109/1983	82/1922	0.77 (0.57 ~ 1.03)	0.075	
Smoke						0.898
No	4449 (57.82)	101/2220	77/2229	0.75 (0.55 ~ 1.02)	0.063	
Yes	3246 (42.18)	140/1950	73/1296	0.77 (0.58 ~ 1.03)	0.082	

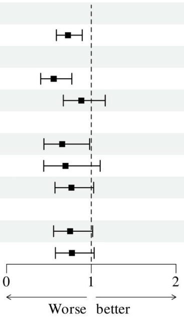

F) Feeling bad about self

Variables	n (%)	Non WCS	WCS	OR (95%CI)	P	P for interaction
		No. of events/ No. of total				
All patients	7695 (100.00)	284/4170	198/3525	0.81 (0.68 ~ 0.98)	0.032	
Gender						0.370
Men	3764 (48.91)	137/2064	85/1700	0.74 (0.56 ~ 0.98)	0.034	
Women	3931 (51.09)	147/2106	113/1825	0.88 (0.68 ~ 1.13)	0.322	
Age						0.296
≤ 35	1911 (24.83)	69/967	57/944	0.84 (0.58 ~ 1.20)	0.334	
≥ 65	1879 (24.42)	68/1220	38/659	1.04 (0.69 ~ 1.56)	0.863	
36-64	3905 (50.75)	147/1983	103/1922	0.71 (0.55 ~ 0.92)	0.009	
Smoke						0.700
No	4449 (57.82)	128/2220	106/2229	0.82 (0.63 ~ 1.06)	0.132	
Yes	3246 (42.18)	156/1950	92/1296	0.88 (0.67 ~ 1.15)	0.344	

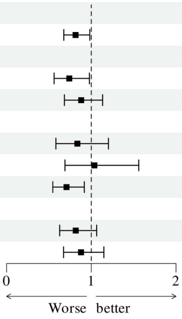

G) Difficulty concentrating

Variables	n (%)	Non WCS	WCS	OR (95%CI)	P	P for interaction
		No. of events/ No. of total				
All patients	7695 (100.00)	186/4170	120/3525	0.75 (0.60 ~ 0.95)	0.019	
Gender						0.279
Men	3764 (48.91)	93/2064	51/1700	0.66 (0.46 ~ 0.93)	0.017	
Women	3931 (51.09)	93/2106	69/1825	0.85 (0.62 ~ 1.17)	0.318	
Age						0.376
≤ 35	1911 (24.83)	41/967	27/944	0.66 (0.41 ~ 1.09)	0.106	
≥ 65	1879 (24.42)	49/1220	27/659	1.02 (0.63 ~ 1.65)	0.932	
36-64	3905 (50.75)	96/1983	66/1922	0.70 (0.51 ~ 0.96)	0.028	
Smoke						0.459
No	4449 (57.82)	76/2220	56/2229	0.73 (0.51 ~ 1.03)	0.074	
Yes	3246 (42.18)	110/1950	64/1296	0.87 (0.63 ~ 1.19)	0.384	

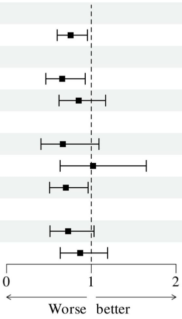

H) Psychomotor disturbance

Variables	n (%)	Non WCS	WCS	OR (95%CI)	P	P for interaction
		No. of events/ No. of total				
All patients	7695 (100.00)	51/4170	22/3525	0.51 (0.31 ~ 0.84)	0.008	
Gender						0.489
Men	3764 (48.91)	26/2064	9/1700	0.42 (0.19 ~ 0.89)	0.024	
Women	3931 (51.09)	25/2106	13/1825	0.60 (0.30 ~ 1.17)	0.133	
Age						0.570
≤ 35	1911 (24.83)	9/967	7/944	0.80 (0.29 ~ 2.14)	0.651	
≥ 65	1879 (24.42)	20/1220	4/659	0.37 (0.12 ~ 1.08)	0.068	
36-64	3905 (50.75)	22/1983	11/1922	0.51 (0.25 ~ 1.06)	0.072	
Smoke						0.203
No	4449 (57.82)	19/2220	14/2229	0.73 (0.37 ~ 1.46)	0.378	
Yes	3246 (42.18)	32/1950	8/1296	0.37 (0.17 ~ 0.81)	0.013	

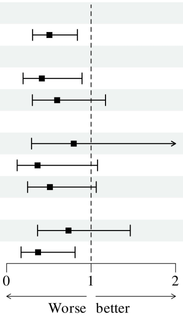

I) Suicidal ideation
