# Supplementary material for: Association between weekend catch-up sleep and specific depressive symptoms: a real world research
Source: Front Psychiatry. 2025 Dec 3;16:1698743. doi: 10.3389/fpsyt.2025.1698743 (PMC12708574; doi:10.3389/fpsyt.2025.1698743)
Supplement: Supplementary file 2 [file DataSheet2.pdf]

Variables	n (%)	Non WCS	WCS	OR (95%CI)	P	P for interaction
		No. of events/ No. of total				
All patients	180 (100.00)	15/41	25/139	0.38 (0.18 ~ 0.82)	0.014	
Gender						0.507
Men	71 (39.44)	7/19	7/52	0.27 (0.08 ~ 0.91)	0.035	
Women	109 (60.56)	8/22	18/87	0.46 (0.17 ~ 1.26)	0.129	
Age						0.921
≤ 35	121 (67.22)	9/20	21/101	0.32 (0.12 ~ 0.88)	0.026	
36-64	59 (32.78)	6/21	4/38	0.29 (0.07 ~ 1.20)	0.087	
Smoke						0.315
No	143 (79.44)	11/28	19/115	0.31 (0.12 ~ 0.76)	0.010	
Yes	37 (20.56)	4/13	6/24	0.75 (0.17 ~ 3.35)	0.706	

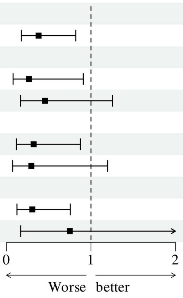

A) Anhedonia

Variables	n (%)	Non WCS	WCS	OR (95%CI)	P	P for interaction
		No. of events/ No. of total				
All patients	180 (100.00)	10/41	15/139	0.38 (0.15 ~ 0.91)	0.031	
Gender						0.826
Men	71 (39.44)	4/19	4/52	0.31 (0.07 ~ 1.40)	0.129	
Women	109 (60.56)	6/22	11/87	0.39 (0.12 ~ 1.20)	0.099	
Age						0.254
≤ 35	121 (67.22)	7/20	11/101	0.23 (0.07 ~ 0.69)	0.009	
36-64	59 (32.78)	3/21	4/38	0.71 (0.14 ~ 3.50)	0.670	
Smoke						0.373
No	143 (79.44)	8/28	12/115	0.29 (0.11 ~ 0.80)	0.017	
Yes	37 (20.56)	2/13	3/24	0.79 (0.11 ~ 5.43)	0.807	

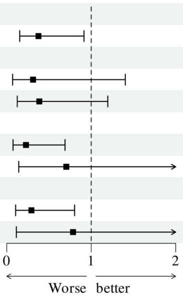

B) Depressed mood

Variables	n (%)	Non WCS	WCS	OR (95%CI)		P	P for interaction
		No. of events/ No. of total					
All patients	180 (100.00)	16/41	39/139	0.61 (0.29 ~ 1.26)		0.183	
Gender							0.478
Men	71 (39.44)	7/19	17/52	0.83 (0.28 ~ 2.50)		0.744	
Women	109 (60.56)	9/22	22/87	0.49 (0.18 ~ 1.30)		0.151	
Age							0.473
≤ 35	121 (67.22)	9/20	28/101	0.47 (0.18 ~ 1.25)		0.131	
36-64	59 (32.78)	7/21	11/38	0.81 (0.26 ~ 2.56)		0.726	
Smoke							0.135
No	143 (79.44)	12/28	29/115	0.45 (0.19 ~ 1.06)		0.068	
Yes	37 (20.56)	4/13	10/24	1.61 (0.38 ~ 6.72)		0.516	

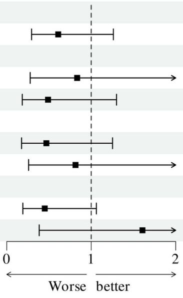

C) Sleep disturbance

Variables	n (%)	Non WCS	WCS	OR (95%CI)		P	P for interaction
		No. of events/ No. of total					
All patients	180 (100.00)	6/41	28/139	1.47 (0.56 ~ 3.84)		0.430	
Gender							0.737
Men	71 (39.44)	2/19	9/52	1.78 (0.35 ~ 9.10)		0.489	
Women	109 (60.56)	4/22	19/87	1.26 (0.38 ~ 4.16)		0.708	
Age							0.239
≤ 35	121 (67.22)	3/20	25/101	1.86 (0.50 ~ 6.89)		0.351	
36-64	59 (32.78)	3/21	3/38	0.51 (0.09 ~ 2.81)		0.443	
Smoke							0.988
No	143 (79.44)	6/28	22/115	0.87 (0.31 ~ 2.39)		0.784	
Yes	37 (20.56)	0/13	6/24	104788671.73 (0.00 ~ Inf)		0.995	

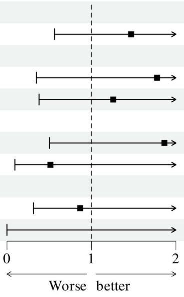

D) Fatigue

Variables	n (%)	Non WCS	WCS	OR (95%CI)	P	P for interaction
		No. of events/ No. of total				
All patients	180 (100.00)	14/41	40/139	0.78 (0.37 ~ 1.64)	0.510	
Gender						0.776
Men	71 (39.44)	6/19	15/52	0.88 (0.28 ~ 2.74)	0.823	
Women	109 (60.56)	8/22	25/87	0.71 (0.26 ~ 1.89)	0.488	
Age						0.150
≤ 35	121 (67.22)	9/20	28/101	0.47 (0.18 ~ 1.25)	0.131	
36-64	59 (32.78)	5/21	12/38	1.48 (0.44 ~ 4.98)	0.529	
Smoke						0.576
No	143 (79.44)	10/28	32/115	0.69 (0.29 ~ 1.66)	0.413	
Yes	37 (20.56)	4/13	8/24	1.12 (0.26 ~ 4.80)	0.874	

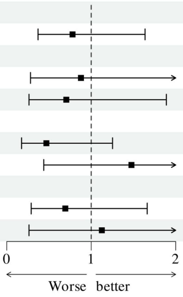

E) Appetite change

Variables	n (%)	Non WCS	WCS	OR (95%CI)	P	P for interaction
		No. of events/ No. of total				
All patients	180 (100.00)	9/41	14/139	0.40 (0.16 ~ 1.00)	0.051	
Gender						0.384
Men	71 (39.44)	2/19	4/52	0.71 (0.12 ~ 4.22)	0.705	
Women	109 (60.56)	7/22	10/87	0.28 (0.09 ~ 0.85)	0.024	
Age						0.572
≤ 35	121 (67.22)	6/20	11/101	0.29 (0.09 ~ 0.89)	0.031	
36-64	59 (32.78)	3/21	3/38	0.51 (0.09 ~ 2.81)	0.443	
Smoke						0.156
No	143 (79.44)	8/28	11/115	0.26 (0.09 ~ 0.74)	0.011	
Yes	37 (20.56)	1/13	3/24	1.71 (0.16 ~ 18.37)	0.656	

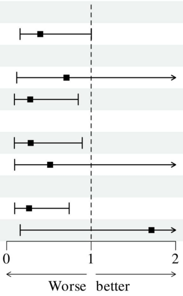

F) Feeling bad about self

Variables	n (%)	Non WCS	WCS	OR (95%CI)	P	P for interaction
		No. of events/ No. of total				
All patients	180 (100.00)	15/41	25/139	0.38 (0.18 ~ 0.82)	0.014	
Gender						0.679
Men	71 (39.44)	6/19	9/52	0.45 (0.14 ~ 1.51)	0.198	
Women	109 (60.56)	9/22	16/87	0.33 (0.12 ~ 0.89)	0.029	
Age						0.546
≤ 35	121 (67.22)	9/20	19/101	0.28 (0.10 ~ 0.78)	0.015	
36-64	59 (32.78)	6/21	6/38	0.47 (0.13 ~ 1.70)	0.249	
Smoke						0.228
No	143 (79.44)	12/28	20/115	0.28 (0.12 ~ 0.68)	0.005	
Yes	37 (20.56)	3/13	5/24	0.88 (0.17 ~ 4.45)	0.874	

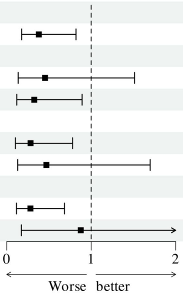

G) Difficulty concentrating

Variables	n (%)	Non WCS	WCS	OR (95%CI)	P	P for interaction
		No. of events/ No. of total				
All patients	7695 (100.00)	186/4170	120/3525	0.75 (0.60 ~ 0.95)	0.019	
Gender						0.279
Men	3764 (48.91)	93/2064	51/1700	0.66 (0.46 ~ 0.93)	0.017	
Women	3931 (51.09)	93/2106	69/1825	0.85 (0.62 ~ 1.17)	0.318	
Age						0.376
≤ 35	1911 (24.83)	41/967	27/944	0.66 (0.41 ~ 1.09)	0.106	
≥ 65	1879 (24.42)	49/1220	27/659	1.02 (0.63 ~ 1.65)	0.932	
36-64	3905 (50.75)	96/1983	66/1922	0.70 (0.51 ~ 0.96)	0.028	
Smoke						0.459
No	4449 (57.82)	76/2220	56/2229	0.73 (0.51 ~ 1.03)	0.074	
Yes	3246 (42.18)	110/1950	64/1296	0.87 (0.63 ~ 1.19)	0.384	

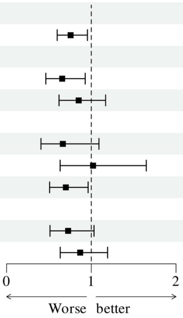

H) Psychomotor disturbance

Variables	n (%)	Non WCS	WCS	OR (95%CI)	P	P for interaction
		No. of events/ No. of total				
All patients	180 (100.00)	8/41	6/139	0.19 (0.06 ~ 0.57)	0.003	
Gender						0.756
Men	71 (39.44)	4/19	2/52	0.15 (0.02 ~ 0.90)	0.038	
Women	109 (60.56)	4/22	4/87	0.22 (0.05 ~ 0.95)	0.042	
Age						0.176
≤ 35	121 (67.22)	6/20	4/101	0.10 (0.02 ~ 0.38)	<.001	
36-64	59 (32.78)	2/21	2/38	0.53 (0.07 ~ 4.05)	0.539	
Smoke						0.399
No	143 (79.44)	7/28	5/115	0.14 (0.04 ~ 0.47)	0.002	
Yes	37 (20.56)	1/13	1/24	0.52 (0.03 ~ 9.10)	0.656	

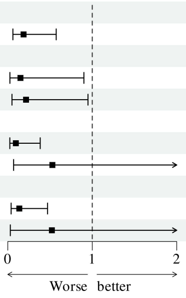

I) Suicidal ideation
