# Supplementary material for: Association between weekend catch-up sleep and specific depressive symptoms: a real world research
Source: Front Psychiatry. 2025 Dec 3;16:1698743. doi: 10.3389/fpsyt.2025.1698743 (PMC12708574; doi:10.3389/fpsyt.2025.1698743)
Supplement: Supplementary file 3 [file DataSheet3.pdf]

Supplementary Table 1: Multivariable Logistic Regression Analysis of the Association Between Weekend Catch-up Sleep and Anhedonia.

	Nhanes		Xijing Hospital	
Variables	*P*	OR (95%CI)	*P*	OR (95%CI)
Intercept	<.001	0.10 (0.07 ~ 0.15)	0.018	7.90 (1.42 ~ 44.05)
WCStwo				
No		1.00 (Reference)		1.00 (Reference)
Yes	0.034	0.84 (0.72 ~ 0.99)	< 0.001	0.18 (0.07 ~ 0.47)
Race				/
Mexican American		1.00 (Reference)		
Other Hispanic	0.976	0.99 (0.71 ~ 1.39)		
Non-Hispanic White	0.789	0.96 (0.73 ~ 1.27)		
Non-Hispanic Black	0.155	1.22 (0.93 ~ 1.62)		
Other Race	0.486	0.89 (0.64 ~ 1.23)		
Education				
Less than high school		1.00 (Reference)		1.00 (Reference)
High school or equivalent	0.246	0.87 (0.68 ~ 1.10)	0.616	0.61 (0.09 ~ 4.24)
College or above	0.020	0.77 (0.62 ~ 0.96)	0.004	0.09 (0.02 ~ 0.46)
Marital				
Married/living with partner		1.00 (Reference)		1.00 (Reference)
Widowed/divorced/separated	0.287	1.11 (0.91 ~ 1.35)	0.009	22.37 (2.21 ~ 226.94)
Never married	0.521	1.07 (0.87 ~ 1.32)	0.011	3.32 (1.32 ~ 8.33)
Obesity (BMI≥30kg/m2)				
No		1.00 (Reference)		1.00 (Reference)
Yes	0.093	1.15 (0.98 ~ 1.35)	0.053	10.05 (0.97 ~ 103.82)
Alcohol				
No		1.00 (Reference)		1.00 (Reference)
Yes	0.059	1.29 (0.99 ~ 1.69)	0.174	0.51 (0.19 ~ 1.35)
Activity				
0 times/week		1.00 (Reference)		1.00 (Reference)
1-2 times/week	0.659	1.07 (0.78 ~ 1.47)	0.045	0.38 (0.15 ~ 0.98)
≥3 times/week	0.985	1.00 (0.71 ~ 1.41)	0.048	0.30 (0.09 ~ 0.99)

Supplementary Table 2: Multivariable Logistic Regression Analysis of the Association Between Weekend Catch-up Sleep and Depressed Mood.

	Nhanes		Xijing Hospital	
Variables	*P*	OR (95%CI)	*P*	OR (95%CI)
Intercept	<.001	0.09 (0.06 ~ 0.14)	0.313	2.59 (0.41 ~ 16.40)
WCStwo				
No		1.00 (Reference)		1.00 (Reference)
Yes	0.010	0.80 (0.67 ~ 0.95)	0.005	0.21 (0.07 ~ 0.62)
Race				
Mexican American		1.00 (Reference)		
Other Hispanic	0.602	0.91 (0.65 ~ 1.28)		
Non-Hispanic White	0.007	0.68 (0.51 ~ 0.90)		
Non-Hispanic Black	0.126	0.80 (0.60 ~ 1.07)		
Other Race	0.288	0.84 (0.61 ~ 1.16)		
Education				
Less than high school		1.00 (Reference)		1.00 (Reference)
High school or equivalent	0.694	1.06 (0.81 ~ 1.38)	0.937	1.09 (0.14 ~ 8.64)
College or above	0.869	1.02 (0.80 ~ 1.30)	0.030	0.15 (0.03 ~ 0.83)
Marital				
Married/living with partner		1.00 (Reference)		1.00 (Reference)
Widowed/divorced/separated	<.001	1.44 (1.18 ~ 1.76)	0.991	0.00 (0.00 ~ Inf)
Never married	0.952	1.01 (0.80 ~ 1.27)	0.030	3.36 (1.12 ~ 10.04)
Obesity (BMI≥30kg/m2)				
No		1.00 (Reference)		1.00 (Reference)
Yes	0.042	1.20 (1.01 ~ 1.42)	0.349	3.28 (0.27 ~ 39.37)
Alcohol				
No		1.00 (Reference)		1.00 (Reference)
Yes	0.095	1.28 (0.96 ~ 1.70)	0.038	0.26 (0.07 ~ 0.93)
Activity				
0 times/week		1.00 (Reference)		1.00 (Reference)
1-2 times/week	0.962	1.01 (0.72 ~ 1.41)	0.110	0.40 (0.13 ~ 1.23)
≥3 times/week	0.894	1.03 (0.71 ~ 1.47)	0.465	0.62 (0.17 ~ 2.24)

Supplementary Table 3: Multivariable Logistic Regression Analysis of the Association Between Weekend Catch-up Sleep and Sleep Disturbance.

	Nhanes		Xijing Hospital	
Variables	*P*	OR (95%CI)	*P*	OR (95%CI)
Intercept	<.001	0.19 (0.14 ~ 0.25)	0.393	1.99 (0.41 ~ 9.65)
WCStwo				
No		1.00 (Reference)		1.00 (Reference)
Yes	0.005	0.84 (0.74 ~ 0.95)	0.116	0.52 (0.23 ~ 1.17)
Race				
Mexican American		1.00 (Reference)		
Other Hispanic	0.495	0.91 (0.71 ~ 1.18)		
Non-Hispanic White	0.573	0.94 (0.76 ~ 1.16)		
Non-Hispanic Black	0.845	0.98 (0.79 ~ 1.21)		
Other Race	0.292	0.88 (0.69 ~ 1.12)		
Education				
Less than high school		1.00 (Reference)		1.00 (Reference)
High school or equivalent	0.833	1.02 (0.84 ~ 1.23)	0.785	0.77 (0.12 ~ 4.86)
College or above	0.227	0.90 (0.76 ~ 1.07)	0.027	0.19 (0.04 ~ 0.82)
Marital				
Married/living with partner		1.00 (Reference)		1.00 (Reference)
Widowed/divorced/separated	<.001	1.29 (1.11 ~ 1.49)	0.117	5.29 (0.66 ~ 42.51)
Never married	0.819	0.98 (0.83 ~ 1.16)	0.054	2.03 (0.99 ~ 4.17)
Obesity (BMI≥30kg/m2)				
No		1.00 (Reference)		1.00 (Reference)
Yes	<.001	1.25 (1.11 ~ 1.42)	0.221	3.65 (0.46 ~ 29.03)
Alcohol				
No		1.00 (Reference)		1.00 (Reference)
Yes	0.047	1.24 (1.01 ~ 1.53)	0.846	0.93 (0.43 ~ 2.00)
Activity				
0 times/week		1.00 (Reference)		1.00 (Reference)
1-2 times/week	0.486	1.09 (0.86 ~ 1.39)	0.964	1.02 (0.45 ~ 2.30)
≥3 times/week	0.859	1.02 (0.79 ~ 1.33)	0.963	0.98 (0.36 ~ 2.65)

Supplementary Table 4: Multivariable Logistic Regression Analysis of the Association Between Weekend Catch-up Sleep and Fatigue.

Variables	*P*	OR (95%CI)	*P*	OR (95%CI)
Intercept	<.001	0.17 (0.12 ~ 0.23)	0.019	9.10 (1.44 ~ 57.57)
WCStwo				
No		1.00 (Reference)		1.00 (Reference)
Yes	0.132	0.91 (0.81 ~ 1.03)	0.138	0.53 (0.23 ~ 1.23)
Race				
Mexican American		1.00 (Reference)		
Other Hispanic	0.886	0.98 (0.76 ~ 1.26)		
Non-Hispanic White	0.691	1.04 (0.85 ~ 1.28)		
Non-Hispanic Black	0.835	1.02 (0.83 ~ 1.27)		
Other Race	0.312	0.88 (0.69 ~ 1.13)		
Education				
Less than high school		1.00 (Reference)		1.00 (Reference)
High school or equivalent	0.162	1.14 (0.95 ~ 1.37)	0.193	0.26 (0.03 ~ 1.99)
College or above	0.095	0.87 (0.73 ~ 1.03)	0.007	0.10 (0.02 ~ 0.53)
Marital				
Married/living with partner		1.00 (Reference)		1.00 (Reference)
Widowed/divorced/separated	0.010	1.21 (1.05 ~ 1.40)	0.656	1.75 (0.15 ~ 20.29)
Never married	0.265	1.09 (0.93 ~ 1.28)	0.103	1.84 (0.88 ~ 3.84)
Obesity (BMI≥30kg/m^2)				
No		1.00 (Reference)		1.00 (Reference)
Yes	<.001	1.30 (1.15 ~ 1.46)	0.286	3.19 (0.38 ~ 26.84)
Alcohol				
No		1.00 (Reference)		1.00 (Reference)
Yes	<.001	1.71 (1.41 ~ 2.07)	0.186	0.57 (0.24 ~ 1.32)
Activity				
0 times/week		1.00 (Reference)		1.00 (Reference)
1-2 times/week	0.528	1.08 (0.85 ~ 1.37)	0.025	0.40 (0.18 ~ 0.89)
≥3 times/week	0.821	1.03 (0.80 ~ 1.33)	0.085	0.41 (0.15 ~ 1.13)

Supplementary Table 5: Multivariable Logistic Regression Analysis of the Association Between Weekend Catch-up Sleep and Appetite Change.

	Nhanes		Xijing Hospital	
Variables	*P*	OR (95%CI)	*P*	OR (95%CI)
Intercept	<.001	0.07 (0.05 ~ 0.10)	0.032	0.07 (0.01 ~ 0.80)
WCStwo				
No		1.00 (Reference)		1.00 (Reference)
Yes	0.793	0.98 (0.84 ~ 1.14)	0.455	1.51 (0.52 ~ 4.39)
Race				
Mexican American		1.00 (Reference)		
Other Hispanic	0.742	1.05 (0.77 ~ 1.44)		
Non-Hispanic White	0.804	0.97 (0.75 ~ 1.25)		
Non-Hispanic Black	0.747	1.04 (0.80 ~ 1.36)		
Other Race	0.990	1.00 (0.74 ~ 1.35)		
Education				
Less than high school		1.00 (Reference)		1.00 (Reference)
High school or equivalent	0.772	1.03 (0.82 ~ 1.30)	0.347	3.32 (0.27 ~ 40.57)
College or above	0.090	0.83 (0.68 ~ 1.03)	0.782	0.73 (0.08 ~ 6.98)
Marital				
Married/living with partner		1.00 (Reference)		1.00 (Reference)
Widowed/divorced/separated	0.001	1.35 (1.12 ~ 1.61)	0.235	4.42 (0.38 ~ 51.48)
Never married	0.299	1.11 (0.91 ~ 1.36)	0.006	3.61 (1.44 ~ 9.02)
Obesity (BMI≥30kg/m^2)				
No		1.00 (Reference)		1.00 (Reference)
Yes	<.001	1.56 (1.34 ~ 1.82)	0.743	1.49 (0.14 ~ 16.00)
Alcohol				
No		1.00 (Reference)		1.00 (Reference)
Yes	0.001	1.50 (1.18 ~ 1.91)	0.302	0.59 (0.22 ~ 1.59)
Activity				
0 times/week		1.00 (Reference)		1.00 (Reference)
1-2 times/week	0.101	1.31 (0.95 ~ 1.81)	0.638	1.27 (0.47 ~ 3.37)
≥3 times/week	0.168	1.28 (0.90 ~ 1.80)	0.374	1.72 (0.52 ~ 5.64)

Supplementary Table 6: Multivariable Logistic Regression Analysis of the Association Between Weekend Catch-up Sleep and Feeling Bad About Self.

	Nhanes		Xijing Hospital	
Variables	P	OR (95%CI)	P	OR (95%CI)
Intercept	<.001	0.05 (0.03 ~ 0.09)	0.990	0.00 (0.00 ~ Inf)
WCStwo				
No		1.00 (Reference)		1.00 (Reference)
Yes	0.002	0.72 (0.58 ~ 0.89)	0.009	0.24 (0.08 ~ 0.70)
Race				
Mexican American		1.00 (Reference)		
Other Hispanic	0.444	1.18 (0.77 ~ 1.79)		
Non-Hispanic White	0.476	0.88 (0.61 ~ 1.26)		
Non-Hispanic Black	0.433	1.16 (0.80 ~ 1.66)		
Other Race	0.765	1.06 (0.71 ~ 1.60)		
Education				
Less than high school		1.00 (Reference)		1.00 (Reference)
High school or equivalent	0.840	0.97 (0.71 ~ 1.32)	0.990	16246458.23 (0.00 ~ Inf)
College or above	0.234	0.84 (0.64 ~ 1.12)	0.990	4697841.84 (0.00 ~ Inf)
Marital				
Married/living with partner		1.00 (Reference)		1.00 (Reference)
Widowed/divorced/separated	0.149	1.20 (0.94 ~ 1.54)	0.128	7.86 (0.55 ~ 112.12)
Never married	0.897	1.02 (0.77 ~ 1.34)	0.217	1.90 (0.69 ~ 5.23)
Obesity (BMI≥30kg/m2)				
No		1.00 (Reference)		1.00 (Reference)
Yes	0.231	1.14 (0.92 ~ 1.40)	0.403	2.80 (0.25 ~ 31.45)
Alcohol				
No		1.00 (Reference)		1.00 (Reference)
Yes	0.035	1.43 (1.02 ~ 1.99)	0.036	0.23 (0.06 ~ 0.91)
Activity				
0 times/week		1.00 (Reference)		1.00 (Reference)
1-2 times/week	0.608	1.12 (0.73 ~ 1.72)	0.195	0.49 (0.17 ~ 1.44)
≥3 times/week	0.220	1.33 (0.84 ~ 2.10)	0.562	0.67 (0.17 ~ 2.59)

Supplementary Table 7: Multivariable Logistic Regression Analysis of the Association Between Weekend Catch-up Sleep and Difficulty Concentrating.

	Nhanes		Xijing Hospital	
Variables	P	OR (95%CI)	P	OR (95%CI)
Intercept	<.001	0.05 (0.03 ~ 0.08)	0.138	3.61 (0.66 ~ 19.66)
WCStwo				
No		1.00 (Reference)		1.00 (Reference)
Yes	0.028	0.81 (0.67 ~ 0.98)	0.002	0.23 (0.09 ~ 0.59)
Race				
Mexican American		1.00 (Reference)		
Other Hispanic	0.529	0.88 (0.60 ~ 1.30)		
Non-Hispanic White	0.050	0.73 (0.53 ~ 0.99)		
Non-Hispanic Black	0.921	1.02 (0.74 ~ 1.39)		
Other Race	0.374	0.85 (0.59 ~ 1.22)		
Education				
Less than high school		1.00 (Reference)		1.00 (Reference)
High school or equivalent	0.395	1.13 (0.85 ~ 1.51)	0.793	0.77 (0.11 ~ 5.33)
College or above	0.489	0.91 (0.70 ~ 1.18)	0.010	0.12 (0.02 ~ 0.60)
Marital				
Married/living with partner		1.00 (Reference)		1.00 (Reference)
Widowed/divorced/separated	0.040	1.27 (1.01 ~ 1.59)	0.162	5.97 (0.49 ~ 73.26)
Never married	0.373	1.12 (0.87 ~ 1.43)	0.009	3.29 (1.34 ~ 8.07)
Obesity (BMI≥30kg/m2)				
No		1.00 (Reference)		1.00 (Reference)
Yes	0.008	1.29 (1.07 ~ 1.55)	0.532	2.20 (0.19 ~ 25.92)
Alcohol				
No		1.00 (Reference)		1.00 (Reference)
Yes	0.707	1.06 (0.77 ~ 1.48)	0.062	0.39 (0.14 ~ 1.05)
Activity				
0 times/week		1.00 (Reference)		1.00 (Reference)
1-2 times/week	0.073	1.46 (0.97 ~ 2.22)	0.567	0.76 (0.30 ~ 1.92)
≥3 times/week	0.236	1.31 (0.84 ~ 2.05)	0.314	0.55 (0.17 ~ 1.77)

Supplementary Table 8: Multivariable Logistic Regression Analysis of the Association Between Weekend Catch-up Sleep and Psychomotor Disturbance.

	Nhanes		Xijing Hospital	
Variables	P	OR (95%CI)	P	OR (95%CI)
Intercept	<.001	0.04 (0.02 ~ 0.08)	0.220	3.12 (0.51 ~ 19.28)
WCStwo				
No		1.00 (Reference)		1.00 (Reference)
Yes	0.021	0.76 (0.60 ~ 0.96)	0.002	0.20 (0.07 ~ 0.57)
Race				
Mexican American		1.00 (Reference)		
Other Hispanic	0.904	0.97 (0.62 ~ 1.52)		
Non-Hispanic White	0.157	0.76 (0.52 ~ 1.11)		
Non-Hispanic Black	0.215	0.78 (0.53 ~ 1.15)		
Other Race	0.148	0.71 (0.45 ~ 1.13)		
Education				
Less than high school		1.00 (Reference)		1.00 (Reference)
High school or equivalent	0.890	1.02 (0.73 ~ 1.44)	0.927	1.10 (0.14 ~ 8.41)
College or above	0.108	0.77 (0.57 ~ 1.06)	0.046	0.18 (0.03 ~ 0.97)
Marital				
Married/living with partner		1.00 (Reference)		1.00 (Reference)
Widowed/divorced/separated	<.001	1.58 (1.21 ~ 2.06)	0.123	7.81 (0.57 ~ 106.24)
Never married	0.925	1.02 (0.73 ~ 1.40)	0.229	1.84 (0.68 ~ 5.01)
Obesity (BMI≥30kg/m2)				
No		1.00 (Reference)		1.00 (Reference)
Yes	0.064	1.25 (0.99 ~ 1.57)	0.990	0.00 (0.00 ~ Inf)
Alcohol				
No		1.00 (Reference)		1.00 (Reference)
Yes	0.990	1.00 (0.66 ~ 1.51)	0.046	0.30 (0.09 ~ 0.98)
Activity				
0 times/week		1.00 (Reference)		1.00 (Reference)
1-2 times/week	0.442	1.21 (0.74 ~ 1.98)	0.206	0.50 (0.17 ~ 1.46)
≥3 times/week	0.326	1.30 (0.77 ~ 2.20)	0.192	0.40 (0.10 ~ 1.58)

Supplementary Table 9: Multivariable Logistic Regression Analysis of the Association Between Weekend Catch-up Sleep and Suicidal Ideation.

	Nhanes		Xijing Hospital	
Variables	P	OR (95%CI)	P	OR (95%CI)
Intercept	<.001	0.01 (0.00 ~ 0.03)	0.994	0.00 (0.00 ~ Inf)
WCStwo				
No		1.00 (Reference)		1.00 (Reference)
Yes	0.007	0.50 (0.30 ~ 0.82)	0.002	0.13 (0.04 ~ 0.46)
Race				
Mexican American		1.00 (Reference)		
Other Hispanic	0.232	1.64 (0.73 ~ 3.70)		
Non-Hispanic White	0.080	0.49 (0.22 ~ 1.09)		
Non-Hispanic Black	0.269	0.63 (0.28 ~ 1.42)		
Other Race	0.983	0.99 (0.42 ~ 2.35)		
Education				
Less than high school		1.00 (Reference)		1.00 (Reference)
High school or equivalent	0.050	2.08 (1.00 ~ 4.33)	0.994	6776492.37 (0.00 ~ Inf)
College or above	0.511	1.27 (0.63 ~ 2.56)	0.994	5603325.99 (0.00 ~ Inf)
Marital				
Married/living with partner		1.00 (Reference)		1.00 (Reference)
Widowed/divorced/separated	0.102	1.56 (0.92 ~ 2.67)	0.996	0.00 (0.00 ~ Inf)
Never married	0.843	0.94 (0.48 ~ 1.81)	0.414	1.67 (0.49 ~ 5.74)
Obesity (BMI≥30kg/m2)				
No		1.00 (Reference)		1.00 (Reference)
Yes	0.249	1.32 (0.82 ~ 2.11)	0.079	10.58 (0.76 ~ 147.21)
Alcohol				
No		1.00 (Reference)		1.00 (Reference)
Yes	0.805	0.90 (0.38 ~ 2.12)	0.568	0.66 (0.16 ~ 2.72)
Activity				
0 times/week		1.00 (Reference)		1.00 (Reference)
1-2 times/week	0.750	0.87 (0.37 ~ 2.04)	0.323	0.51 (0.13 ~ 1.94)
≥3 times/week	0.674	0.82 (0.32 ~ 2.10)	0.127	0.23 (0.03 ~ 1.52)
